# Detecting subclinical cardiotoxicity during immune checkpoint inhibitor therapy: a combined GLS and ECG repolarization analysis

**DOI:** 10.3389/fonc.2025.1615209

**Published:** 2025-07-08

**Authors:** Dogac Oksen, Muhammed Heja Gecit, Sukru Arslan, Muzaffer Aslan, Yunus Emre Yavuz, Saban Secmeler, Veysel Oktay

**Affiliations:** ^1^ Department of Cardiology, Altinbas University Medical Faculty, Istanbul, Türkiye; ^2^ Department of Cardiology, Istanbul University – Cerrahpasa Institute of Cardiology, Istanbul, Türkiye; ^3^ Department of Cardiology, Siirt University Medical Faculty, Siirt, Türkiye; ^4^ Department of Cardiology, Necmettin Erbakan University Medical Faculty, Konya, Türkiye; ^5^ Department of Medical Oncology, Altinbas University Medical Faculty, Istanbul, Türkiye

**Keywords:** immune checkpoint inhibitors, cardiotoxicity, cardio-oncology, global longitudinal strain, repolarization abnormalities

## Abstract

**Purpose:**

Immune checkpoint inhibitors (ICIs) can induce subclinical cardiac dysfunction that often goes undetected by conventional monitoring. This study evaluated whether echocardiographic global longitudinal strain (GLS) and electrocardiographic (ECG) repolarization parameters could detect early, subclinical cardiotoxicity in patients with cancer and without pre-existing cardiovascular disease undergoing ICI therapy.

**Methods:**

A observational cohort study included 74 patients with cancer treated with ICIs between January 2023 and August 2024. Echocardiographic GLS measurements and detailed ECG analyses were performed at baseline and repeated at 6 months. Cardiotoxicity was defined as a significant reduction in GLS or left ventricular ejection fraction. Correlations between GLS and ECG repolarization parameters were statistically assessed.

**Results:**

At 6 months, significant subclinical myocardial impairment was observed, with GLS decreasing from 19.40 ± 1.07% to 17.70 ± 1.62% (p<0.001). Notable ECG changes included increased QT dispersion (40.10 ± 10.55 ms to 50.20 ± 15.30 ms, p=0.001), QTc prolongation (420.45 ± 20.60 ms to 430.75 ± 25.40 ms, p=0.013), increased Tp-e interval (80.21 ± 10.45 ms to 85.30 ± 12.40 ms, p=0.021), and elevated heart rate (72.21 ± 8.40 bpm to 75.35 ± 9.15 bpm, p=0.037). GLS was negatively correlated with QT dispersion (r = -0.845, p < 0.05) and Tp-e intervals (r = -0.478, p = 0.052).

**Conclusion:**

GLS and ECG repolarization parameters, particularly QT dispersion and Tp-e intervals, effectively detect subclinical myocardial dysfunction in patients with cancer undergoing ICI therapy. These findings underscore the importance of comprehensive cardiac monitoring to enable early intervention and mitigate cardiotoxicity risk during immunotherapy.

## Graphical Abstract

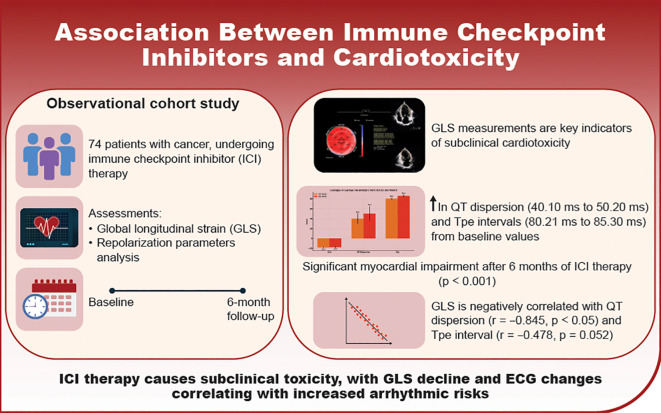

## Introduction

Immunotherapies have revolutionized cancer treatment, becoming a cornerstone of modern therapeutic strategies by enabling the immune system to recognize and target cancer cells more effectively (1). Among these, immune checkpoint inhibitors (ICIs) have significantly improved survival and quality of life across various cancer types. ICIs act by targeting key immune regulatory pathways, such as cytotoxic T-lymphocyte-associated protein 4 (CTLA-4), programmed cell death protein 1 (PD-1), and programmed death ligand 1 (PD-L1), thereby enhancing the immune system’s ability to detect and eliminate cancer cells (2).

Despite their efficacy, ICIs can trigger a broad activation of the immune system, leading to a distinct spectrum of side effects known as immune-related adverse events (irAEs). These can affect multiple organ systems, including the cardiovascular system. ICIs have been associated with myocarditis, driven by excessive T-cell-mediated inflammation, and acute coronary syndrome linked to destabilization of atherosclerotic plaques (1, 3). Other reported irAEs include vasculitis, pericarditis, and arrhythmia, which can range from subclinical manifestations to life-threatening complications (4).

As clinical symptoms emerge, managing these adverse events becomes increasingly difficult. Therefore, early detection of myocardial dysfunction at a subclinical level is crucial to initiating cardioprotective treatment and minimizing exposure to the causative agent. Conventional methods often fail to identify irAEs in their early stages before clinical symptoms appear. However, global longitudinal strain (GLS)—a widely available echocardiographic tool—offers detailed insights into myocardial deformation, enabling the early detection of subclinical cardiac injury (5). Current guidelines strongly recommend GLS, particularly in patients receiving potentially cardiotoxic therapies (6).

Additionally, electrocardiographic (ECG) abnormalities, such as T-wave anomalies, ST-segment changes, QT interval prolongation, and increased QT dispersion, have been observed in the early stages of ICI therapy. Since ECG changes are commonly associated with myocarditis, detailed analysis of repolarization parameters has shown promise in identifying early myocardial injury before clinical symptoms manifest (7).

This article explores the cardiac impacts of ICIs, focusing on how changes in GLS and ECG repolarization parameters can serve as early indicators of myocardial stress or damage. By recognizing these subtle yet critical alterations, clinicians may be able to implement timely interventions, ultimately enhancing the cardiovascular safety profile of these transformative cancer therapies. This integrative approach enhances our understanding of ICI-related cardiotoxicity and highlights the importance of interdisciplinary collaboration in the era of oncologic immunotherapy.

## Materials and methods

### Study population and design

This observational cohort study was conducted at our center’s oncology clinic, enrolling patients scheduled to initiate immunotherapy who had no prior history of cardiovascular disease. The study spanned from January 2023 to August 2024, with the final 6-month follow-up assessments completed in February 2025. Eligible participants were adults (≥18 years) of any gender diagnosed with malignancies treated with ICI. Individuals were excluded if they had a history of coronary and/or peripheral artery disease, confirmed atherosclerosis, previous myocarditis or pericarditis, reduced ventricular systolic function at any time on echocardiography, impaired strain parameters, moderate to severe valve stenosis or insufficiency, arrhythmias, or prior use of oral anticoagulants, antiplatelet, or antiarrhythmic drugs for any reason. Additionally, patients who failed to attend regular follow-ups chose not to participate, or had inconsistent medication use were not included in the study.

At enrollment, all participants underwent a baseline cardiovascular evaluation, including ECG, conventional echocardiography, and advanced tissue deformation analysis to assess myocardial function. These assessments were repeated 6 months after immunotherapy initiation. Patient characteristics and demographic data were obtained from medical records and patient histories. The primary objective was to identify subclinical cardiac dysfunction associated with immunotherapy by analyzing changes in ECG repolarization parameters, GLS from echocardiography, and other structural and functional cardiac indices. This approach facilitated early detection and management of potential cardiotoxic effects, aiming to enhance treatment safety and patient outcomes.

Cardiotoxicity was defined as a ≥10% decrease in left ventricular ejection fraction (LVEF), an LVEF <53%, or a >15% relative reduction in GLS compared to baseline (6). The study received ethical approval from the Istanbul University Cerrahpasa Ethics Committee (2025/195 approval number assigned on January 2, 2025) and adhered to the Declaration of Helsinki guidelines for clinical research. Written informed consent was obtained from all participants.

### Immunotherapy administration

Immunotherapy was administered as part of the oncological treatment regimen for all participants in this study. The choice of immune checkpoint inhibitors, such as PD-1, PD-L1, and CTLA-4 inhibitors, was determined based on tumor type and applicable clinical guidelines at the time of treatment (8). Dosing schedules followed product labeling and oncological standards of care, typically involving cycles every 2 to 6 weeks, depending on the specific agent and clinical context.

Before initiating therapy, all patients underwent a comprehensive oncological and medical evaluation to confirm their suitability for immunotherapy. This assessment included tumor staging, a review of prior cancer treatments, and a detailed medical history to identify any contraindications. Throughout the treatment period, oncologists closely monitored patients for immune-related adverse events, adjusting treatment protocols as needed to manage emerging side effects. This proactive approach optimized therapeutic outcomes while minimizing potential risks associated with immunotherapy.

### ECG evaluation

ECG was performed as part of the initial cardiovascular assessment and at the 3-month follow-up for all study participants to monitor and detect any immunotherapy-induced changes in cardiac electrical activity. Standard 12-lead ECGs were obtained using a high-resolution electrocardiograph while patients were at rest. The recordings analyzed various parameters, including heart rate, rhythm, PR interval, QRS duration, QT interval, QT dispersion, and Tp-e interval.

The QT interval was assessed using the tangent method, which involves tracing a line from the onset of the QRS complex to the endpoint of the T-wave, primarily in lead II. If the T-wave termination was unclear in lead II, measurements were taken from leads V5 or V6. To standardize the QT interval relative to heart rate, we performed correction using Bazett’s formula QTcB = QT/√RR), which adjusts the QT interval by dividing it by the square root of the RR interval, ensuring accurate correction in relevant cases (9, 10).

Special attention was given to repolarization parameters, including the ST segment, T-wave anomalies, and the Tp-e/QT ratio, which are critical for detecting early signs of myocardial injury or dysfunction. All ECG analyses were conducted by certified cardiologists blinded to the patient’s clinical status. This methodological rigor ensured accurate and unbiased evaluation of ECG changes, providing a reliable assessment of the cardiotoxic effects of immunotherapy.

### Echocardiographic evaluation

Comprehensive echocardiographic assessments were performed at baseline and at the 6-month follow-up to evaluate the impact of immunotherapy on cardiac function. The evaluations included conventional echocardiography, GLS and LVEF measurements using Simpson’s method. Two experienced investigators conducted the assessments using a Philips EPIC CVx ultrasound system (Holland) equipped with 3 MHz and 7 MHz cardiac probes. During echocardiographic examinations, participants were positioned in the left lateral decubitus position, and heart rhythm was continuously monitored via a single-lead ECG. To enhance accuracy, we recorded the mean of three sequential heartbeats.

Echocardiographic imaging included comprehensive views, such as parasternal, apical four- and five-chamber, and subcostal views. The dimensions of the left and right atria and the left ventricle’s end-diastolic and end-systolic sizes were measured from the parasternal long-axis view. Right ventricular baseline dimensions were assessed from the apical four-chamber view. LVEF was calculated using Simpson’s method. All procedures adhered to the American Society of Echocardiography guidelines and the European Association of Cardiovascular Imaging (11).

**Figure 1:**
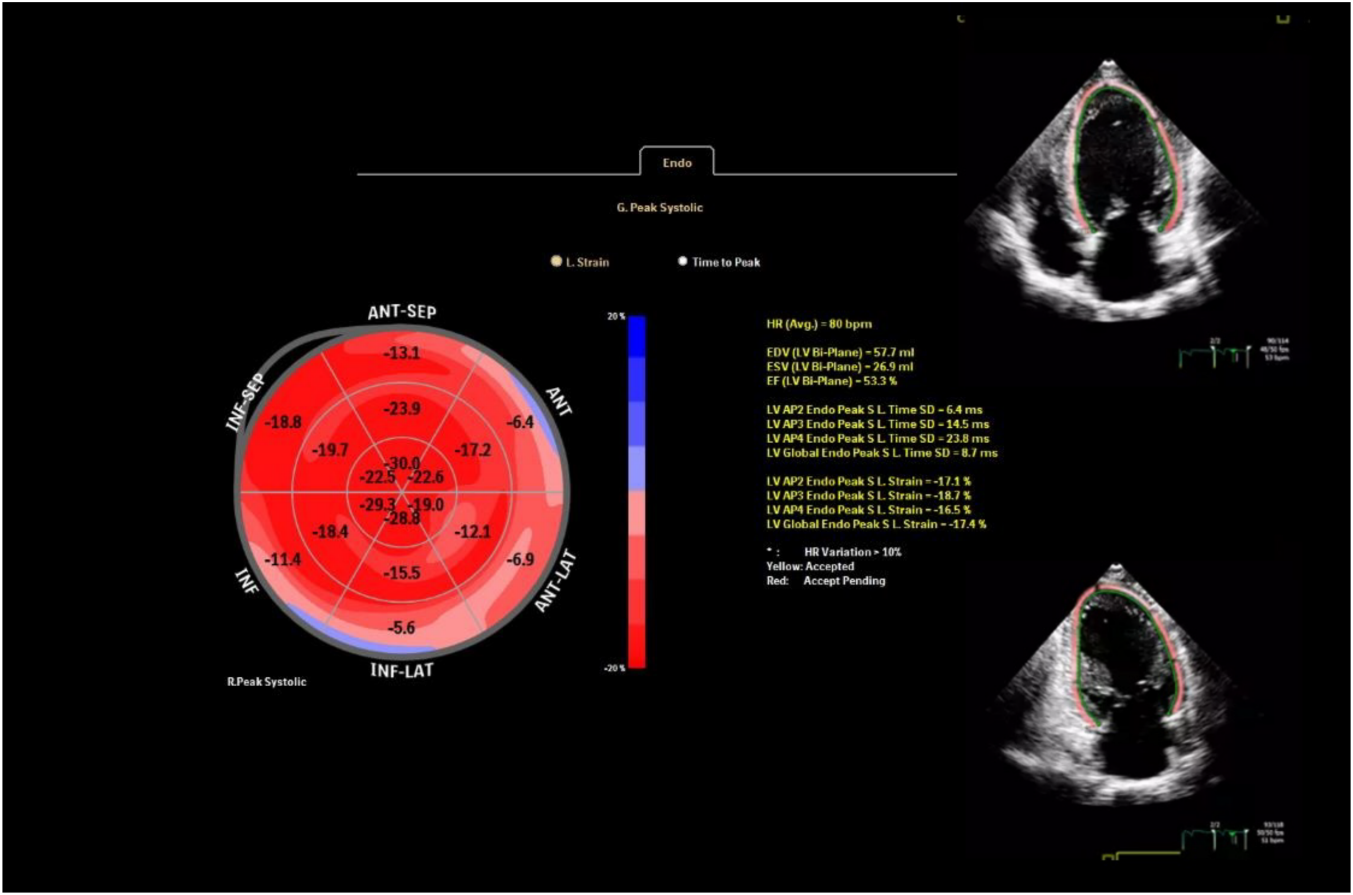
Measurement of global longitudinal strain using speckle-tracking echocardiography.

GLS was derived using speckle-tracking echocardiography analyzed from the apical four-, two-, and three-chamber views with AutoStrain (Philips, Munich, Germany). To ensure privacy, we anonymized all echocardiographic images before analysis. GLS values were obtained by averaging the segmental strains across all 17 myocardial segments (**Figure 1**). For precise two-dimensional speckle-tracking analysis of myocardial deformation, settings such as depth, image sector, and frame rate were carefully optimized. The frame rate was maintained between 50 and 70 frames per second to ensure high-quality imaging and accurate tracking of myocardial speckles throughout the cardiac cycle. A mean GLS change of ~1.5% with SD ~2.0%, a sample size of 74 provided >80% power (β=0.2) to detect a 1.5% change in GLS at α=0.05.

### Reproducibility

To assess the reliability of our measurements, we used intraclass correlation coefficients (ICC) to evaluate both intraindividual and interobserver variations. ECG and echocardiographic data from 10 randomly selected patients were reanalyzed by the original observer to minimize intraindividual variability. These same datasets and corresponding images were independently evaluated by a second observer to assess interobserver variability. These steps ensured the robustness and reproducibility of our findings by quantifying measurement consistency across different assessments and observers within the study.

### Statistical analysis

Statistical analysis was conducted using SPSS software (version 25.0, IBM Corp., Armonk, NY, USA). Descriptive statistics were used to summarize the demographic and clinical characteristics of the participants. Continuous variables were expressed as means ± standard deviations, while categorical variables were presented as frequencies and percentages.

For the primary analysis, repeated-measures ANOVA was used to assess changes in GLS and echocardiographic parameters over time, specifically comparing baseline values with those obtained at the 6-month follow-up. Assumptions of sphericity were tested using Mauchly’s test, and the Greenhouse-Geisser correction was applied where necessary. Data normality was assessed using the Shapiro–Wilk test. When data did not follow a normal distribution, Spearman’s rank correlation was used for a nonparametric analysis of relationships. Pearson’s correlation coefficients were calculated to determine the strength and direction of correlations. A two-tailed p-value of <0.05 was considered statistically significant for all analyses.

## Results

### Study population

This prospective cohort study initially included 92 participants. However, six patients had previously received cardiotoxic treatment, five with coronary artery disease or heart failure, and seven were lost to follow-up, leaving 74 eligible participants. The median follow-up duration was 180 days (interquartile range [IQR]: 150–210 days). The median time from initial cancer diagnosis to the initiation of ICI therapy was 7.5 months (IQR: 4.0–13.0 months). The study population consisted of 27% females (n=20), with a mean age of 64.8 ± 12.4 years. Detailed demographic characteristics, cardiovascular risk factors, cancer types, and administered ICI are summarized in **Tables 1**, **2**.

**Table 1:** Baseline characteristics of study participants.

Variables	Mean ± S.D. (n = 74)
Age, years	64.8 ± 12.4
Female gender, % (n)	27.0 (20)
BMI, kg/m^2^	26.4 [19.5–33.8]
Cardiovascular risk factors	
Current smoking, % (n)	35.1 (26)
Hypertension, % (n)	31.0 (23)
Diabetes mellitus, % (n)	17.5 (13)
COPD, % (n)	16.2 (12)
Chronic kidney disease, % (n)	10.8 (8)
Follow-up duration, days [IQR]	180.0 [165–220]

COPD, chronic obstructive pulmonary disease.

**Table 2:** Baseline characteristics: primary cancer types and immune checkpoint inhibitor regimens.

Variables	Mean ± S.D. (n = 74)
Primary cancer type	
Melanoma, % (n)	12.1 (9)
Lung cancer, % (n)	37.8 (28)
Renal cell carcinoma, % (n)	12.1 (9)
Breast cancer, % (n)	16.2 (12)
Head and neck cancer, % (n)	6.8 (5)
Other, % (n)	14.8 (11)
Immune checkpoint inhibitors	
Anti-PD-1, nivolumab	45.9 (34)
Anti-PD-1, pembrolizumab	25.6 (19)
Anti-CTLA-4, ipilimumab	5.4 (4)
Anti-PD-L1	13.5 (10)
Combination of nivolumab and ipilimumak	9.4 (7)

PD-1, programmed cell death protein 1; CTLA-4, cytotoxic T-lymphocyte-associated protein 4; PD-L1, programmed death ligand protein 1.

### Cardiotoxicity and echocardiographic findings

**Figure 2:**
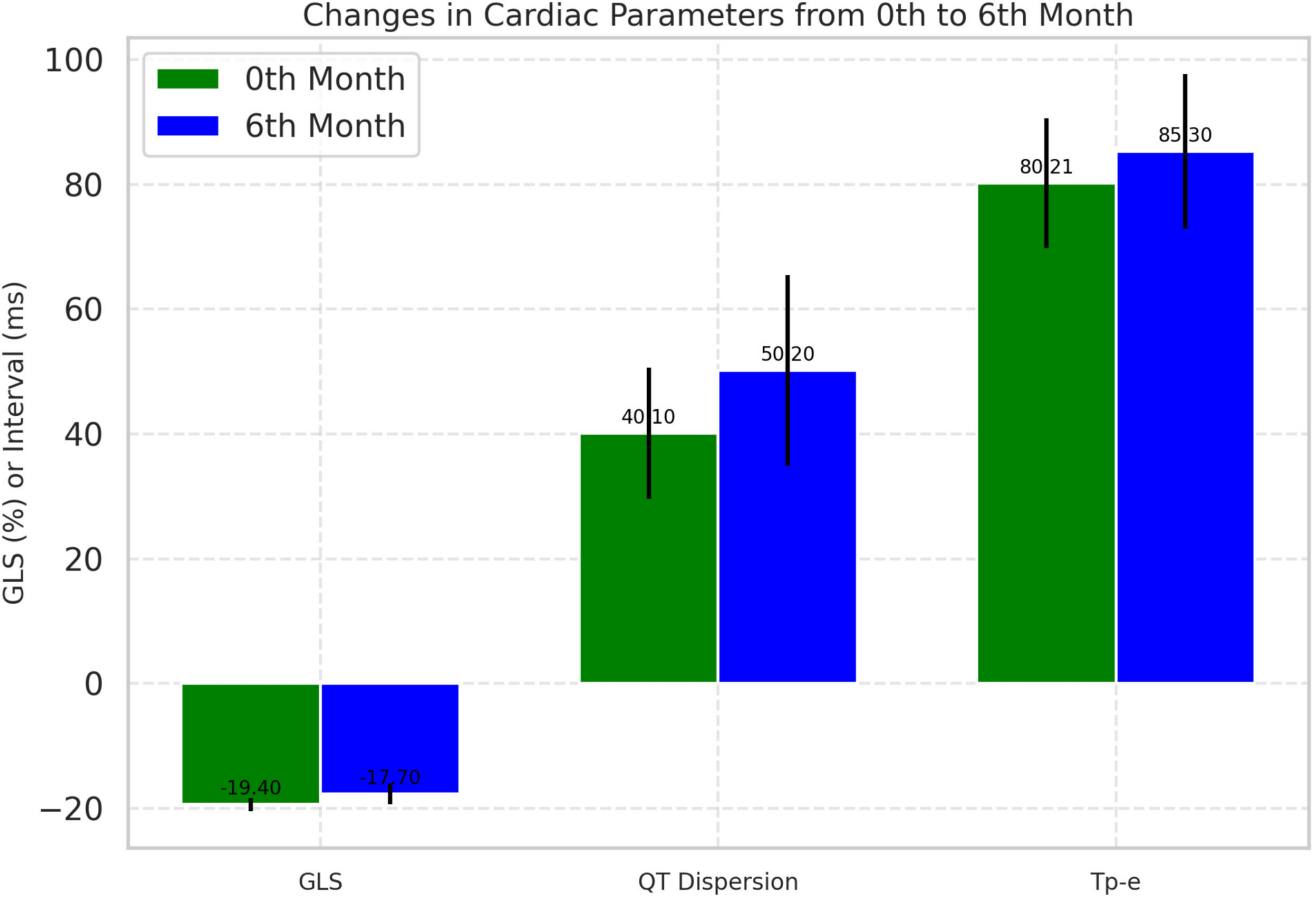
Comparative analysis of cardiac parameters at baseline and 6-month follow-up. Changes in global longitudinal strain and QT dispersion from baseline to 6-month follow-up. Box plots represent median, IQR, and outliers. GLS, Global longitudinal strain.

During the follow-up, cardiotoxicity, defined as an LVEF <53% and a >10% decrease in LVEF, was observed in two patients. Although the mean LVEF declined from 63.13 ± 2.08 to 62.00 ± 2.48, this change was insignificant (p=0.280). Conventional echocardiographic and tissue Doppler parameters are presented in **Table 3**. GLS significantly decreased at the 6-month follow-up compared to baseline (19.40 ± 1.07 *vs*. 17.70 ± 1.62; p < 0.001), indicating a notable impact of ICI treatment on myocardial function (**Figure 2**).

**Table 3:** Echocardiographic parameters before and after ICI treatment.

Variables	Pre-treatment	Post-treatment	P-value
LVEDd, mm	46.13 ± 5.10	47.47 ± 4.69	0.065
LVESd, mm	25.58 ± 4.76	26.49 ± 5.43	0.222
LVEDv, mL	72.00 ± 12.96	80.19 ± 10.41	0.062
LVESv, mL	33.23 ± 3.12	35.60 ± 2.07	0.324
LVEF, %	63.13 ± 2.08	62.00 ± 2.48	0.280
LA, mm	34.60 ± 8.40	36.32 ± 9.54	0.333
E/e’	7.50 ± 0.27	8.20 ± 0.23	0.407
MeanEm (cm/s)	8.31 ± 1.28	8.13 ± 1.51	0.562
MeanAm (cm/s)	10.48 ± 2.24	10.55 ± 2.72	0.865
MeanEm/Am ratio	0.84 ± 0.20	0.81 ± 0.25	0.055
E/E’(Em)	8.79 ± 2.27	8.27 ± 2.25	0.572
GLS, %	19.40 ± 1.07	17.70 ± 1.62	<0.001

LVEDd, left ventricular end-diastolic diameter; LVESd, left ventricular end-systolic diameter; LVEDv, left ventricular end-diastolic volume; LVEDs, left ventricular end-systolic volume; LVEF, left ventricular ejection fraction; LA, left atrium; E/e’, ratio of early mitral inflow velocity to early diastolic mitral annular velocity; GLS, global longitudinal strain.

### ECG changes

At baseline, all participants (100%) were in sinus rhythm, which slightly decreased to 98.6% post-treatment, though this change was not statistically significant. Significant ECG changes included an increase in mean heart rate from 72.21 ± 8.40 bpm to 75.35 ± 9.15 bpm (p=0.037), QTc prolongation from 420.45 ± 20.60 ms to 430.75 ± 25.40 ms (p=0.013), and an increase in QT dispersion from 40.10 ± 10.55 ms to 50.20 ± 15.30 ms (p=0.001). The Tp-e interval, which reflects the transmural dispersion of repolarization across the ventricular wall, increased from 80.21 ms at baseline to 85.30 ms at 6 months (p= 0.021) (**Figure 1**). Additionally, the Tp-e/QT ratio increased significantly from 0.190 to 0.200 (p = 0.044) (**Table 4**).

**Table 4:** Electrocardiographic parameters before and after ICI treatment.

Variables	Pre-treatment	Post-treatment	P value
Sinus rhythm, % (n)	100.0 (74)	98.6 (73)	N/A
Heart rate, beats/min	72.21 ± 8.40	75.35 ± 9.15	0.037
PR interval, ms	160.40 ± 12.50	162.55 ± 13.30	0.198
QRS duration, ms	90.50 ± 12.30	92.40 ± 13.85	0.158
QTc interval, ms	420.45 ± 20.60	430.75 ± 25.40	0.013
QT dispersion, ms	40.10 ± 10.55	50.20 ± 15.30	0.001
Tp-e interval, ms	80.21 ± 10.45	85.30 ± 12.40	0.021
Tp-e/QTc ratio	0.190 ± 0.020	0.200 ± 0.021	0.044

cQT, corrected QT interval; Tp-e, T peak-to-end interval.

### Correlation between GLS and ECG parameters

**Figure 3:**
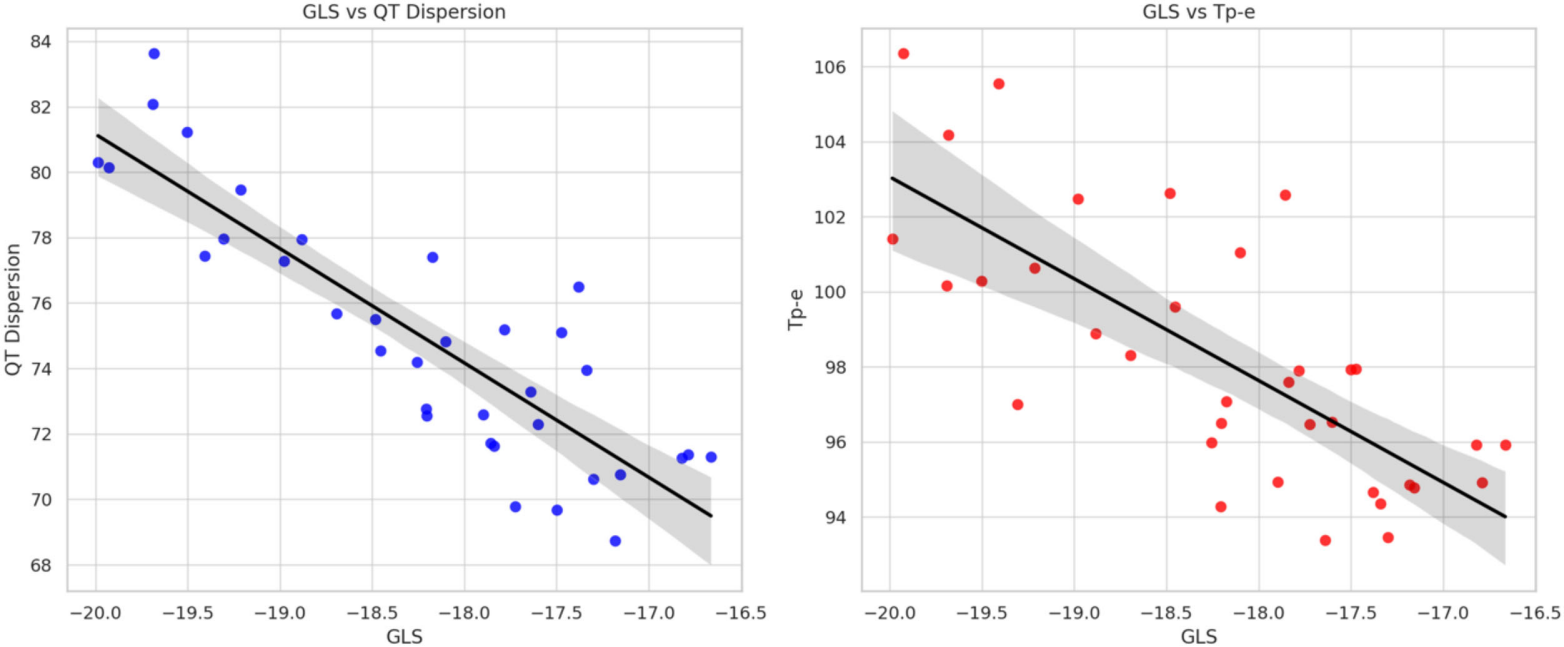
Correlation between global longitudinal strain, QT dispersion, and T peak-to-end interval during immune checkpoint inhibitor therapy. Scatter plot showing the correlation between GLS and QT dispersion (left), and Tp-e interval (right). Pearson correlation coefficients are shown. GLS, Global longitudinal strain.

Significant correlations were observed between GLS and ECG repolarization parameters, critical for assessing ICI-induced cardiotoxicity. GLS showed a strong negative correlation with QT dispersion (r = -0.845, p < 0.05). A moderate negative correlation was also observed between changes in GLS and Tp-e intervals (r = -0.478, p = 0.052) (**Figure 3**).

## Discussion

This study provides valuable insights into the cardiotoxic effects associated with ICIs. Consistent with previous research, our findings demonstrate a significant impact on cardiac parameters, particularly GLS, QT dispersion, and Tp-e intervals. The significant decrease in GLS observed at the 6-month follow-up suggests potential myocardial stress or damage induced by ICIs, aligning with the findings of Awadalla et al., who reported similar GLS reductions following ICI therapy (12). Additionally, our analysis identified significant correlations between GLS and various ECG repolarization parameters, which are crucial for assessing ICI-induced cardiotoxicity. Specifically, a significant negative correlation was observed between GLS and both the Tp-e interval and QT dispersion.

ICIs are designed to enhance the immune response against cancer cells by blocking inhibitory pathways through monoclonal antibodies, thereby promoting cytotoxic T lymphocyte-mediated cell death (13). However, this immune activation can lead to cardiovascular adverse events, including severe, life-threatening conditions such as fulminant myocarditis, myopericarditis, cardiac dysfunction, arrhythmias, and/or myocardial infarction. In a case series on ICI-induced myocarditis, the reported mortality rate reached 50% within a median of 30 days after symptom onset (14).

Current cardio-oncology guidelines recommend routine monitoring with transthoracic echocardiography and, when feasible, GLS assessment for patients undergoing cardiotoxic chemotherapy. However, monitoring strategies for ICI therapy differ, with initial evaluations in high-risk patients, including ECG, troponin levels, and NT-proBNP testing, followed by ongoing surveillance throughout treatment. In cases where myocarditis is suspected, further assessments such as transthoracic echocardiography to evaluate LVEF and GLS, as well as cardiac MRI, are recommended (6).

GLS is a highly sensitive imaging technique that can detect myocardial damage at an early stage and provide an accurate assessment of cardiac functions. Compared to LVEF, GLS is particularly effective in identifying subclinical ventricular dysfunction (15). Its high sensitivity has established GLS as a reproducible and widely accessible imaging modality for evaluating myocardial effects caused by non-cardiac diseases and treatments. Additionally, careful and skilled use of transthoracic echocardiographic imaging can achieve excellent clinical outcomes without requiring additional resources.

Few studies have utilized strain echocardiography in patients undergoing ICI therapy. Awadalla et al. compared 101 patients who developed myocarditis during ICI therapy with 92 control patients receiving the same treatment and having similar demographic characteristics. The groups were identical in gender, cancer type, and age. While no decrease in LVEF was observed in patients with myocarditis, a decline in GLS was noted. In contrast, no significant change in GLS was observed in patients who did not develop myocarditis after treatment. The rate of major adverse cardiac events (MACE) was 4.4 times higher in patients with a decrease in LVEF and 1.4 times higher in those with a decrease in GLS (12).

Coskun et al. compared post-treatment LVEF and GLS in 44 patients receiving ICI therapy, with a median follow-up of 5.3 months. While no significant reduction in LVEF was observed, a statistically significant decline in GLS was reported, consistent with our findings. Notably, our study included a larger patient cohort and a longer follow-up period, yet the results are broadly consistent with the study (16). Mirza et al. compared strain echocardiography results of eight patients undergoing cardiac MRI during ICI therapy with those of healthy individuals. LVEF remained above 50% in all participants. Despite preserved conventional LVEF, the mean GLS in the ICI-treated group was significantly lower than in the control group (ICI: −12.381 ± 4.161; control: −19.761 ± 1.925; p < 0.001) (17). Another study on ICI-associated cardiotoxicity found that GLS declined as the duration of ICI therapy increased. While no significant changes were observed at 3 months compared to baseline, a statistically significant reduction in GLS was noted at 6 months (p<0.05). However, no significant changes were detected in conventional echocardiographic parameters or LVEF. Similarly, our study found no differences between baseline and 6 months in conventional echocardiographic parameters, tissue Doppler, or LVEF measured using the Simpson method, yet GLS decreased significantly. This finding highlights the superiority of GLS over conventional LVEF in detecting subclinical cardiac dysfunction. Speckle-tracking imaging is crucial in the early quantitative assessment of myocardial functions. Furthermore, our observations are in accordance with current cardio-oncology guidelines, which advocate for the routine use of GLS and ECG-based parameters for the early detection of cardiotoxicity in patients receiving cancer therapies (6). These collective findings highlight the superior sensitivity of GLS and ventricular repolarization indices in identifying early myocardial dysfunction. They also support the integration of multimodal cardiac imaging and ECG monitoring as a cornerstone of contemporary cardio-oncology surveillance strategies.

Along with ICI therapy, irAEs were reported by Johnson et al. in 2016, who described cases of fulminant myocarditis and later published a retrospective report on its incidence (18). Further data from public databases indicate an increased frequency of myocardial damage associated with ICI therapy (19).

ECG is a first-line, widely accessible tool for cardiac assessment. Despite its simplicity, it provides highly valuable information. During ICI therapy, ECG changes related to myocardial involvement have been reported. Myocardial damage associated with ICIs and ECG abnormalities has been documented in 72 case reports and six clinical studies. ST-T changes, low voltage, and prolonged QRS and QT intervals have been linked to myocardial injury (20).

A retrospective review of 73 patients with ICI-associated myocarditis found that those with severe myocarditis had significantly longer QT intervals, QRS durations exceeding 110 ms, bundle branch blocks, and sinus tachycardia compared to those with milder cases. Early ECG changes have been reported as potential predictors of severe myocarditis (21).

Among cardiovascular irAEs, repolarization abnormalities are the most frequently reported. QT prolongation and increased QT dispersion can occur depending on the dose and duration of ICI therapy. These repolarization abnormalities have been observed to correlate directly with elevated troponin and NT-proBNP levels (22). Increased QT dispersion and Tp-e intervals are particularly concerning, as they are associated with a higher risk of arrhythmic events. Our findings align with those of Wang et al., who reported that increased QT dispersion may indicate an elevated risk of adverse cardiac events in patients undergoing cancer therapy (23). These ECG changes suggest that ICIs may exacerbate underlying myocardial repolarization heterogeneity, thereby increasing the risk of arrhythmias.

The observed association between GLS and QT dispersion may reflect a shared pathophysiological pathway involving myocardial inflammation and electrical heterogeneity, which is increasingly recognized in ICI-related myocarditis. A decrease in GLS, indicative of subclinical myocardial dysfunction, was significantly associated with an increase in QT dispersion—a marker of heightened heterogeneity in ventricular repolarization and a known predictor of arrhythmic risk. In addition, lower GLS values tended to correlate with prolonged Tp-e intervals, which reflect transmural dispersion of repolarization. However, the Tp-e/QTc ratio, another proposed index of repolarization heterogeneity, did not show a statistically significant correlation with GLS in this study. Coskun et al. evaluated ECG-based parameters such as Tp-e and QT dispersion in cancer patients undergoing ICI therapy but did not observe significant changes over time or clear predictive value for arrhythmias within a limited follow-up period and small cohort size (24).

These ECG changes emphasize the importance of regular cardiac monitoring in patients receiving ICIs, highlighting the need for careful management strategies to mitigate the potential risks of significant cardiotoxic effects. Furthermore, these findings suggest that GLS, combined with detailed ECG analysis, could serve as a critical component of cardiovascular risk assessment in this patient population, aiding in therapeutic decision-making and monitoring strategies in oncological care. Incorporating GLS and ECG markers into routine cardiotoxicity monitoring may enable oncologists and cardiologists to detect myocardial stress early, guide treatment modifications, and improve patient outcomes during immunotherapy.

Although subgroup differences could be clinically meaningful, the sample size limited statistical power for such analyses. This warrants further investigation in multicenter datasets. Future studies with larger populations should include regression-based models adjusting for confounders such as age, cancer type, baseline comorbidities, and type/duration of ICI therapy. Despite its limitations, the study design incorporates robust methodology including blinded analyses, reproducibility assessment, and standardized definitions of subclinical cardiotoxicity, thereby enhancing its internal validity.

## Limitations

The primary limitation of this study is the lack of clinical correlation between echocardiographic and ECG assessments. No MACE occurred during the 6-month follow-up period, preventing the collection of statistically significant data; therefore, they were not included. Extending the follow-up period or increasing the patient population could address this limitation. Due to the observational design and lack of a control group, causal inference is limited, and the cardiotoxic changes observed may also be influenced by concurrent clinical factors Another key limitation is the absence of cardiac biomarkers, such as Nt-proBNP and troponin. These biomarkers were not included due to limited data availability resulting from financial constraints. Additionally, although deformation analysis was performed using echocardiography, circumferential and radial strains were excluded due to prolonged procedure times and challenges in obtaining optimal echocardiographic windows.

## Conclusion

Despite their substantial clinical benefits, ICI carries a significant risk of cardiotoxicity, which may initially present without clear symptoms. Our findings highlight the importance of echocardiographic GLS and ECG repolarization parameters, particularly QT dispersion and Tp-e intervals, as sensitive tools for the early detection of subclinical myocardial dysfunction during ICI therapy. Incorporating these parameters into routine clinical practice may facilitate the timely recognition of cardiotoxic effects, enabling earlier intervention and potentially improving patient outcomes. Future research with larger patient cohorts, including regression-based models that adjust for confounders, is needed to validate these findings and refine cardiovascular monitoring protocols for patients undergoing immunotherapy.

## Data Availability

The raw data supporting the conclusions of this article will be made available by the authors, without undue reservation.
